# Effect of differentiation, *de novo* innervation, and electrical pulse stimulation on mRNA and protein expression of Na^+^,K^+^-ATPase, FXYD1, and FXYD5 in cultured human skeletal muscle cells

**DOI:** 10.1371/journal.pone.0247377

**Published:** 2021-02-26

**Authors:** Vid Jan, Katarina Miš, Natasa Nikolic, Klemen Dolinar, Metka Petrič, Andraž Bone, G. Hege Thoresen, Arild C. Rustan, Tomaž Marš, Alexander V. Chibalin, Sergej Pirkmajer

**Affiliations:** 1 Institute of Pathophysiology, Faculty of Medicine, University of Ljubljana, Ljubljana, Slovenia; 2 Section for Pharmacology and Pharmaceutical Biosciences, Department of Pharmacy, University of Oslo, Oslo, Norway; 3 Department of Pharmacology, Institute of Clinical Medicine, University of Oslo, Oslo, Norway; 4 National Research Tomsk State University, Tomsk, Russia; 5 Department of Molecular Medicine and Surgery, Integrative Physiology, Karolinska Institutet, Stockholm, Sweden; Universidade Federal do Rio de Janeiro, BRAZIL

## Abstract

Denervation reduces the abundance of Na^+^,K^+^-ATPase (NKA) in skeletal muscle, while reinnervation increases it. Primary human skeletal muscle cells, the most widely used model to study human skeletal muscle *in vitro*, are usually cultured as myoblasts or myotubes without neurons and typically do not contract spontaneously, which might affect their ability to express and regulate NKA. We determined how differentiation, *de novo* innervation, and electrical pulse stimulation affect expression of NKA (α and β) subunits and NKA regulators FXYD1 (phospholemman) and FXYD5 (dysadherin). Differentiation of myoblasts into myotubes under low serum conditions increased expression of myogenic markers CD56 (NCAM1), desmin, myosin heavy chains, dihydropyridine receptor subunit α_1S_, and SERCA2 as well as NKAα2 and FXYD1, while it decreased expression of FXYD5 mRNA. Myotubes, which were innervated *de novo* by motor neurons in co-culture with the embryonic rat spinal cord explants, started to contract spontaneously within 7–10 days. A short-term co-culture (10–11 days) promoted mRNA expression of myokines, such as IL-6, IL-7, IL-8, and IL-15, but did not affect mRNA expression of NKA, FXYDs, or myokines, such as musclin, cathepsin B, meteorin-like protein, or SPARC. A long-term co-culture (21 days) increased the protein abundance of NKAα1, NKAα2, FXYD1, and phospho-FXYD1^Ser68^ without attendant changes in mRNA levels. Suppression of neuromuscular transmission with α-bungarotoxin or tubocurarine for 24 h did not alter NKA or FXYD mRNA expression. Electrical pulse stimulation (48 h) of non-innervated myotubes promoted mRNA expression of NKAβ2, NKAβ3, FXYD1, and FXYD5. In conclusion, low serum concentration promotes NKAα2 and FXYD1 expression, while *de novo* innervation is not essential for upregulation of NKAα2 and FXYD1 mRNA in cultured myotubes. Finally, although innervation and EPS both stimulate contractions of myotubes, they exert distinct effects on the expression of NKA and FXYDs.

## Introduction

Skeletal muscle fibers are excited to contract by the α-motor neurons via the neuromuscular junctions. Excitation of skeletal muscle fibers displaces Na^+^ and K^+^ across the membrane, which promotes loss of muscle excitability and contractility [1]. Ion displacement during contractions is opposed by Na^+^,K^+^-ATPase (NKA), which pumps Na^+^ ions out of and K^+^ ions into the cell [2–4]. Capacity of skeletal muscle to perform ion transport depends on the total NKA content (reviewed in [5, 6]), which is reduced by denervation [7]. Reinnervation increases NKA content [8], but whether innervation exerts differential effects on NKA isozymes and the regulatory FXYD proteins is not known.

NKA isozymes are heterodimers comprising the α-subunit (α1–4 isoforms) and the glycoprotein β-subunit (β1–3 isoforms) [9]. Different combinations of isoforms give rise to NKA isozymes with distinct functions and tissue distributions [9–11]. NKAα1 is the most abundant α-isoform in immature skeletal muscle, but NKAα2 is upregulated during postnatal myogenesis [12] and ultimately represents 60–90% of α-subunits (reviewed in [5]). NKAα2 is thought to be particularly important during contractions, while NKAα1 is supposed to primarily maintain ion transport at rest [13], although it may also participate in the process of muscle hypertrophy [14]. Whether innervation differentially regulates expression of the NKAα subunits has not been established.

FXYD proteins (FXYD1-7 in mammals) are small transmembrane proteins that regulate NKA [15]. FXYD1 (phospholemman) and FXYD5 (dysadherin) are the most abundant FXYDs in skeletal muscle [16]. FXYD1 inhibits NKA by reducing its affinity for Na^+^ [11, 17], which can be reversed by phosphorylation of FXYD1 at Ser^63^ and Ser^68^ [10, 11, 18, 19], while FXYD5 increases *V*_*max*_ of NKA [10, 20]. In skeletal muscles of persons with spinal cord injury, content of FXYD1 is reduced, while FXYD5 is increased [16], indicating a role for innervation in regulation of their expression. However, in the spinal cord injury skeletal muscle remains innervated by the α-motor neurons, whose role in regulation of FXYD1 and FYXD5 is unknown.

Differentiated primary human skeletal muscle cells (myotubes), the most widely used model to study human skeletal muscle *in vitro* [21, 22], are usually cultured without neurons. Unlike in human skeletal muscle tissue, in which NKAα2 is by far the most prominently expressed NKAα isoform [23], NKAα1 predominates in aneurally cultured myotubes [24]. Innervation can be established *de novo* by co-culturing myotubes with explants of the embryonic rat spinal cord [25], which leads to formation of functional neuromuscular junctions, maturation of contractile apparatus, and spontaneous contractions of myotubes ([25], reviewed in [26]). Contractions are inhibited by antagonists of the nicotinic acetylcholine receptor (nAChR), such as tubocurarine, α-bungarotoxin, and rocuronium [25, 27], indicating that contractions in this model are driven by motor neurons. Since denervation reduces and reinnervation increases the abundance of NKA in skeletal muscle under *in vivo* conditions [7, 8], we hypothesized that innervation of cultured myotubes might increase NKA content and lead to more mature expression pattern of NKAα subunits.

Aneurally cultured myotubes not only lack innervation, but also typically do not contract unless exposed to electrical pulse stimulation (EPS) [28–30]. NKA content in skeletal muscle is increased by various types of exercise training and decreased by chronic physical inactivity (reviewed in: [5, 6, 31]), indicating contractile activity might affect NKA expression in cultured myotubes independently of innervation. We therefore hypothesized that EPS-evoked contractions might promote NKA expression in otherwise quiescent non-innervated myotubes. To test these possibilities we determined how differentiation under low serum conditions, *de novo* innervation, and EPS affected expression of NKA subunits, FXYD1, and FXYD5 in cultured human skeletal muscle cells.

## Methods

### Ethics evaluation

The skeletal muscle tissue for preparation of human skeletal muscle cell cultures was obtained with informed written consent, signed by the participants or (in the case of minors) their legal representatives, and with approval by the Republic of Slovenia National Medical Ethics Committee (*m*. *semitendinosus*, permit numbers 71/05/12 and 0120-698/2017/4) and by the Regional Committee for Medical and Health Research Ethics South East, Oslo, Norway (*m*. *obliquus internus abdominis*, permit number S-04133). The *vastus lateralis* biopsies were obtained after informed written consent and approval by the Regional Committee for Medical and Health Research Ethics North, Tromsø, Norway (reference number: 2011/882). All experiments were conducted in accordance with the Declaration of Helsinki and Good Laboratory Practice regulations. Experiments with co-cultures with the embryonic rat spinal cord were conducted according to the 3R principle, meaning that the number of rats used and their suffering were minimized. The procedures involving the use of rats and their tissues, were approved by the Ethics Committee for Experiments on Animals at the Administration for Food Safety, Veterinary Sector and Plant Protection (U34401-38/2015/6 and U34401-2/2020/4) and by the National Animal Research Authority, Mattilsynet, Norway (FOTS identification number 6620).

### Materials

Rat skeletal muscle cell line L6, used for evaluation of species specificity of gene expression assays, was from ATCC (Manassas, VA, U.S.). Cell culture flasks, plates and petri dishes were from Sarstedt (Nümbrecht, Germany) or TPP (Trasandingen, Switzerland). Advanced MEM (#12492), DMEM-Glutamax^TM^ (5.5 mM glucose), fetal bovine serum (FBS), Fungizone (250 μg/mL of amphotericin B), gentamicin (10 mg/mL), α-bungarotoxin (#B1601), Pierce BCA Protein Assay Kit, Pierce 660nm Protein Assay Reagent, Pierce Enhanced Chemiluminescence (ECL) Western Blotting Substrate, High-Capacity cDNA Reverse Transcription Kit, and TaqMan Universal Master Mix were from Thermo Fisher Scientific (Waltham, MA, U.S.). Adhesive films and 96-well reaction plates were either from Thermo Fisher Scientific (Waltham, MA, U.S.) or Bio-Rad (Hercules, CA, U.S.). RNeasy Mini Plus Kit and RNeasy Fibrous Tissue Mini Kit were from Qiagen (Hilden, Germany). E.Z.N.A. HP Total RNA Kit was from Omega Bio-Tek (Norcross, GA, U.S.). Ham’s F14 medium was from Biowest SAS (Nuaillé, France) or Thermo Fisher Scientific (Waltham, MA, U.S.). 4–12% Criterion XT Bis-Tris polyacrylamide gels, XT MES electrophoresis buffer, goat anti-rabbit IgG—horseradish peroxidase conjugate (#170–6515) and goat anti-mouse IgG—horseradish peroxidase conjugate (#170–6516) were from Bio-Rad (Hercules, CA, U.S.). Amersham ECL Full-Range Rainbow Molecular Weight Markers were from GE Healthcare Life Sciences (Uppsala, Sweden). Polyvinylidene fluoride (PVDF) membrane, gelatine, fibroblast growth factor, epidermal growth factor, insulin and tubocurarine (#T2379) were from Merck/Sigma-Aldrich (Darmstadt, Germany). MACS CD56 microbeads were from Miltenyi Biotec (Bergisch Gladbach, Germany). CP-BU NEW X-ray films were form AGFA HealthCare (Mortsel, Belgium). All other reagents, unless otherwise specified, were from Merck and of analytical grade. Gene expression assays and primary antibodies are listed in designated sections.

### Culturing and differentiation of human skeletal muscle cells

Primary human skeletal muscle cells for differentiation and co-culture (innervation) experiments were prepared from samples of the *semitendinosus* muscles, which were obtained during routine orthopaedic surgery as surgical waste, and cultured as described [24, 30, 32]. All donors of skeletal muscle tissue required surgery for medical reasons and no tissue was removed solely for the purpose of research. Briefly, human skeletal muscle cells were cultured in growth medium (Advanced MEM with 10% (v/v) FBS, 0.3% (v/v) Fungizone (0.75 μg/mL amphotericin B), and 0.15% (v/v) gentamicin (15 μg/mL) in humidified atmosphere with 5% (v/v) CO_2_. Before reaching confluence, muscle cells were separated into the CD56^+^ and CD56^-^ fractions using MACS CD56 microbeads and cultured in growth medium until the 3^rd^ passage. Gene expression and protein abundance in non-differentiated skeletal muscle cells were analysed before fusion into myotubes. CD56^+^ and CD56^-^ cells were differentiated for 7 days in Advanced MEM with 2% (v/v) FBS.

### Co-cultures of human myotubes with the embryonic rat spinal cord

Co-cultures were prepared from human skeletal muscle cells isolated from the *semitendinosus* muscle as described [25, 26, 30]. CD56^+^ cells were cultured in 6-well plates, coated with 1:2 mixture of 1.5% gelatine and human plasma, in growth medium for 6 days and then in Ham’s F14 medium with FBS (10% (v/v)), fibroblast growth factor (50 ng/mL), epidermal growth factor (10 ng/mL), and insulin (10 μg/mL) for 24 h according to the standard procedure [27, 33, 34]. Explants of the embryonic rat spinal cord, isolated from embryos of pregnant Wistar rats, which were sacrificed in a CO_2_ chamber on the 14^th^ day of gestation, were positioned on human skeletal muscle cells. Co-cultures and aneural controls without the explants were grown in F14 medium with FBS (10% (v/v)) and insulin (10 μg/mL) for 10–11 days (short-term co-cultures) or 21–22 days (long-term co-cultures) according to the standard protocol [25–27, 30, 33, 34].

### Electrical Pulse Stimulation (EPS) of human myotubes

Human skeletal muscle cell cultures used to assess effects of EPS on expression of NKA subunits and FXYDs were established as described [29, 30]. Human satellite cells were obtained from tissue samples of the *obliquus internus abdominis* or the *vastus lateralis* muscles of healthy volunteers, using previously described methods [35]. Briefly, myoblasts were cultured in growth medium DMEM-Glutamax^TM^ (5.5 mM glucose) supplemented with 10% FBS, 25 IU penicillin, 25 μg/ml streptomycin, 1.25 μg/ml amphotericin B, 50 ng/ml gentamicin. At approximately 80% confluency, the growth medium was replaced by DMEM-Glutamax (5.5 mM glucose) supplemented with 2% FBS, 25 IU penicillin, 25 μg/ml streptomycin, 1.25 μg/ml amphotericin B, 50 ng/ml gentamicin, and 25 pM insulin. Multinucleated myotubes grown on Corning CellBind 6-well plates (Corning, Life-Sciences, Schiphol-Rijk, The Netherlands) were submitted to EPS (single, bipolar pulses of 2 ms, 30 V and 1 Hz) by using the 6-well plate culture dish carbon electrode system (IonOptix, Dublin, Ireland). The myotubes were stimulated continuously for the last 48 h of the 7 days differentiation period as previously described [29]. During EPS, differentiation medium was changed every 24 h. Unstimulated (control) myotubes were cultured in the same way as those exposed to EPS.

### Quantitative real-time PCR (qPCR)

The total RNA was extracted from cells with RNeasy Mini Plus Kit or E.Z.N.A. HP Total RNA Kit, and from tissues with RNeasy Fibrous Tissue Mini Kit. Reverse transcription was performed using the High-Capacity cDNA Reverse Transcription Kit. qPCR was performed on ABI PRISM SDS 7500 using TaqMan Gene Expression Assays (Thermo Fisher Scientific, Waltham, MA, U.S.): NCAM1 (CD56, Hs00941830_m1), desmin (Hs00157258_m1), CACNA1S (Hs00163885_m1), MYH1 (Hs00428600_m1), MYH2 (Hs00430042_m1), MYH7 (Hs01110632_m1), ATP2A1 (Hs04983082_m1), ATP2A2 (Hs05000494_m1), ATP2A3 (Hs00545433_g1), FXYD1 (Hs00245327_m1 and Hs00964977_g1), FXYD5 (Hs00204319_m1), ATP1A1 (Hs00167556_m1), ATP1A2 (Hs00265131_m1 and Hs01560077_m1), ATP1A3 (Hs00958036_m1 and Hs00958043_mH), ATP1B1 (Hs00426868_g1), ATP1B2 (Hs05015165_s1), ATP1B3 (Hs00740857_mH), IL6 (Hs00985639_m1 and Hs00174131_m1), IL7 (Hs00174202_m1), CXCL8 (IL8, Hs00174103_m1), IL15 (Hs01003716_m1), OSTN (Hs00898258_m1), CTSB (Hs00947433_m1), METRNL (Hs00417150_m1), SPARC (Hs00234160_m1), 18S rRNA (Hs99999901_s1), cyclophilin (PPIA) (Hs99999904_m1), and β-actin (ACTB) (Hs99999903_m1). Results are reported as gene expression ratios: (1+*E_reference_*)*^Ct,reference^*/(1+*E_target_*)*^Ct,target^*, where E is the PCR efficiency and C_t_ is the threshold cycle [36, 37]. The geometric mean of two endogenous controls (18S rRNA, ACTB, or PPIA) was used to calculate the gene expression ratios for all experiments except for validation of PCR assays and analysis of gene expression in the short-term (10–11 days) co-cultures, as indicated in figure legends. Analysis of gene expression in the short-term co-cultures was performed with only one endogenous control due to insufficient amount of samples.

### Immunoblotting

The cells were washed with ice-cold phosphate-buffered saline (PBS: 137 mM NaCl, 2.7 mM KCl, 10 mM Na_2_HPO_4_, 1.8 mM KH_2_PO_4_, pH 7.4) and harvested with the Laemmli buffer (62.5 mM Tris-HCl (pH 6.8), 2% (w/v) sodium dodecyl sulfate (SDS), 10% (w/v) glycerol, 5% (v/v) 2-mercaptoethanol, 0.002% (w/v) bromophenol blue), as previously described [32]. Protein concentration was measured with Pierce 660 nm Protein Assay.

Proteins were resolved with SDS-PAGE (4–12% polyacrylamide gels) at 200 V using XT MES buffer and transferred to PVDF membrane at 100 V in transfer buffer (31 mM Tris, 0.24 M glycine, 10% (v/v) methanol and 0.01% (w/v) SDS) using the Criterion system (Bio-Rad (Hercules, CA, U.S.). Loading and transfer were evaluated by Ponceau S (0.1% (w/v) in 5% (v/v) acetic acid) staining. Membranes were blocked with 7.5% (w/v) dry milk in Tris-buffered saline with Tween (TBST: 20 mM Tris, 150 mM NaCl, 0.02% (v/v) Tween 20, pH 7.5) for 1 h at room temperature. After blocking, membranes were incubated with primary antibodies (in 20 mM Tris, 150 mM NaCl, pH 7.5, 0.1% (w/v) BSA and 0.1% (w/v) NaN_3_) overnight at 4°C. Membranes were subsequently washed with TBST and incubated with secondary antibody-horseradish peroxidase conjugate in TBST with 5% (w/v) dry milk for 1 h at room temperature. Immunoreactive bands were visualized on X-ray films using enhanced chemiluminescence and quantified using GS-800 Densitometer and Quantity One 1-D Analysis Software 4.6.8. (Bio-Rad, Hercules, CA, U.S.).

Antibodies NKAα1 (05–369) and NKAα2 (AB9094) were from Merck (Darmstadt, Germany), phospho-FXYD1^Ser68^ (PAB0389) from Abnova (Taipei, Taiwan), FXYD1 (13721-1-AP) from Proteintech (Rosemont, IL, U.S.), Sp1 (D4C3, 9389) from Cell Signaling Technology (Danvers, MA, U.S.), and actin (sc1616r) from Santa Cruz Biotechnology (Dallas, TX, U.S.). Antibody against FXYD5 was a kind gift from Dr. Haim Garty (Weizmann Institute of Science, Rehovot, Israel) [38]. SC-71 antibody which detects fast MyHC-2A in rats [39], but both MyHC-2A and 2X in humans [40, 41], and BA-D5 antibody (detects MyHC-β/slow) [42] were a kind gift from Dr. Marija Meznarič and Dr. Vika Smerdu (Faculty of Medicine, University of Ljubljana). SC-71 and BA-D5 antibodies were produced from hybridoma cell lines (Deutsche Sammlung von Mikroorganismen und Zellkulturen, Braunschweig, Germany). Further information on used primary antibodies can be found in Table 1.

**Table 1:** Primary antibodies used in the present study.

Antibody	Host	Type	Dilution	Supplier	Cat. No.
NKAα1	Mouse	Monoclonal	1:2000	Merck	05–369
NKAα2	Rabbit	Polyclonal	1:4000	Merck	AB9094
pFXYD1^Ser68^	Rabbit	Polyclonal	1:2000	Abnova	PAB0389
FXYD1	Rabbit	Polyclonal	1:500	Proteintech	13721-1-AP
Sp1 (D4C3)	Rabbit	Monoclonal	1:1000	CST	9389
Actin	Rabbit	Polyclonal	1:1500	SCB	sc1616r
FXYD5	Mouse	Polyclonal	1:500	See text.	
SC-71	Mouse	Monoclonal	1:150	See text.	
BA-D5	Mouse	Monoclonal	1:250	See text.	

CST: Cell Signaling Technology; SCB: Santa Cruz Biotechnology.

### Statistical analysis

Data are presented as means ± SEM. Statistical analysis was performed using GraphPad Prism 6 (GraphPad Software, San Diego, CA, U.S.). Significance (*P*<0.05) was determined using two-way ANOVA with Bonferroni’s *post hoc* test for multiple comparisons or Student’s ratio paired t-test for comparison of two groups. The number of replicates (n) refers to the number of donors (number of independent human skeletal muscle cell cultures obtained from different donors).

## Results

### Regulation of ion transport machinery during differentiation of CD56+ and CD56- cells

**Fig 1:**
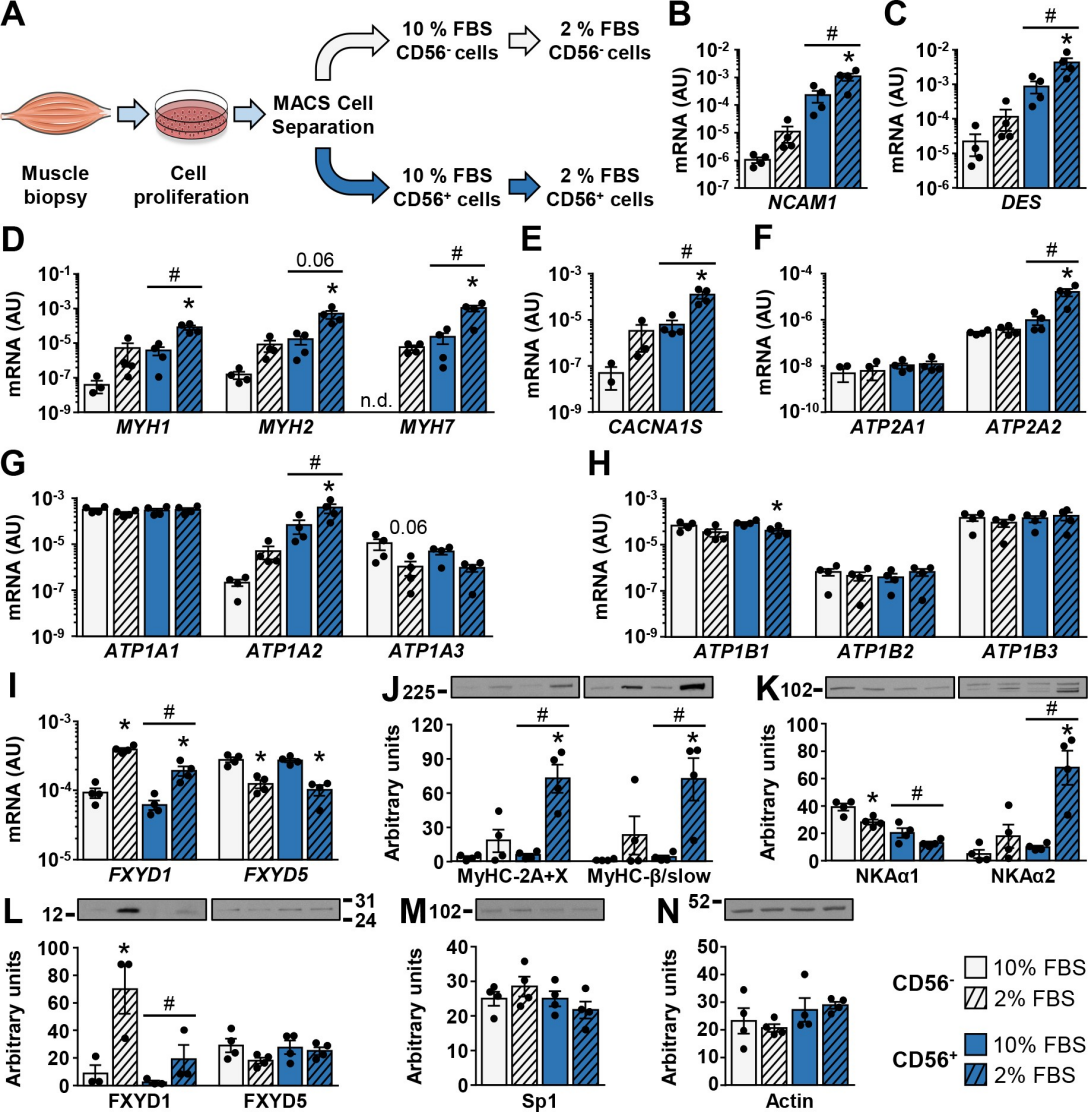
Regulation of ion transport machinery during differentiation of CD56^+^ and CD56^-^ cells. (A) Human skeletal muscle cells were isolated from samples of *m*. *semitendinosus* and grown in Advanced MEM with 10% FBS. Before reaching confluence, cells were separated into CD56^+^ and CD56^-^ fractions using MACS microbeads. CD56^+^ and CD56^-^ cells were subsequently grown in Advanced MEM with 10% FBS (CD56^+^ 10% FBS and CD56^-^ 10% FBS cells) or were differentiated for 7 days in Advanced MEM with 2% FBS (CD56^-^ 2% FBS and CD56^+^ 2% FBS cells). (B-I) qPCR was used to estimate gene expression of (B) *NCAM1* (CD56), (C) desmin, (D) myosin heavy chains *MYH1*, *MYH2*, and *MYH7*, (E) *CACNA1S*, (F) SERCA1 (*ATP2A1*) and SERCA2 (*ATP2A2*), (G) NKAα1–3 (*ATP1A1-3*), (H) NKAβ1–3 (*ATP1B1-3*), and (I) *FXYD1* and *FXYD*5. A geometric mean of 18S rRNA and cyclophilin (PPIA) was used for normalization of gene expression. (J-N) Immunoblotting was used to estimate protein abundance of (J) myosin heavy chains MyHC-2A and -2X, and MyHC-β/slow, (K) NKAα1 and NKAα2, (L) FXYD1 and FXYD5, (M) Sp1, and (N) actin. Note that in humans, SC-71 antibody detects MyHC-2A and -2X (see Methods). Results are means ± SEM (n = 3–4), *P<0.05 vs. 10% FBS, #*P*<0.05 vs CD56.

To enrich the population of myogenic cells, we separated primary human skeletal muscle cells into two fractions based on expression of CD56 (aka neural adhesion molecule 1, NCAM1) [43–45] (Fig 1A and 1B). Differentiation was induced by incubating cells under low-serum (2% FBS) conditions for 7 days. To normalize the expression of target genes, 18S rRNA and cyclophilin (PPIA) were used as endogenous controls.

Desmin (Fig 1C), an intermediary filament characteristic of myogenic cells [46], myosin heavy chains MyHC-2X (gene *MYH1*), MyHC-2A (gene *MYH2*), and MyHC-β/slow (gene *MYH7*) (Fig 1D and 1J), and the α_1S_ subunit of the dihydropyridine receptor (DHPRα_1S_, *CACNA1S*) (Fig 1E), a voltage-gated Ca^2+^ channel that is located in T-tubules of muscle cells [47], were markedly higher in CD56^+^ cells and were further upregulated after culturing in 2% FBS. A similar trend was observed in CD56^-^ cells, although the differences between high and low serum conditions did not reach statistical significance (Fig 1C–1E and 1J). Expression of the sarco(endo)plasmic reticulum Ca^2+^-ATPase SERCA1 (*ATP2A1*) was similar between CD56^+^ and CD56^-^ cells and serum conditions (Fig 1F). In contrast, SERCA2 (*ATP2A2*) was higher in CD56^+^ cells and was upregulated in 2% FBS (Fig 1F). SERCA3 (*ATP1A3*) was below the detection limit under all conditions.

As assessed by measuring mRNA, NKAα1 (*ATP1A1*) was the major NKAα subunit in CD56^+^ and CD56^-^ cells (Fig 1G). Differentiation in 2% FBS tended to reduce the protein abundance of NKAα1 in CD56^+^ (*P* = 0.0912) and CD56^-^ (*P*<0.05) cells (Fig 1K) without major changes in mRNA expression (Fig 1G). NKAα2 (*ATP1A2*) was more prominently expressed in CD56^+^ cells and was markedly upregulated during differentiation (Fig 1G and 1K). Conversely, NKAα3 (*ATP1A3*) mRNA, which was expressed at a very low level under all experimental conditions, tended to be downregulated in differentiated CD56^+^ cells (Fig 1G). However, expression levels of NKAα3 mRNA were low and NKAα3 protein could not be reliably quantified with immunoblot, suggesting these alterations might not be functionally important.

The mRNA expression of NKAβ subunits was similar between CD56^+^ and CD56^-^ cells (Fig 1H). Differentiation reduced gene expression of NKAβ1 (*ATP1B1*) in CD56^+^ cells without major changes in expression of NKAβ2 (*ATP1B2*) and NKAβ3 (*ATP1B3*) (Fig 1H). FXYD1 was more prominently expressed in CD56^-^ than CD56^+^ cells (Fig 1I and 1L). Differentiation markedly upregulated FXYD1 mRNA and protein in CD56^-^ cells, with a similar trend in CD56^+^ cells (Fig 1I and 1L). Despite mRNA expression, FXYD1 protein was detected only in cells from three out of four donors. The mRNA expression of FXYD5 was reduced without significant changes in its protein abundance during differentiation of CD56^+^ and CD56^-^ cells (Fig 1I and 1L).

Finally, the abundance of transcription factor Sp1 (Fig 1M), which is known to regulate expression of various NKA subunits [48], and of actin (Fig 1N) was similar between different cell types and serum conditions.

### Validation of qPCR assays for estimation of gene expression in innervated human myotubes

**Fig 2:**
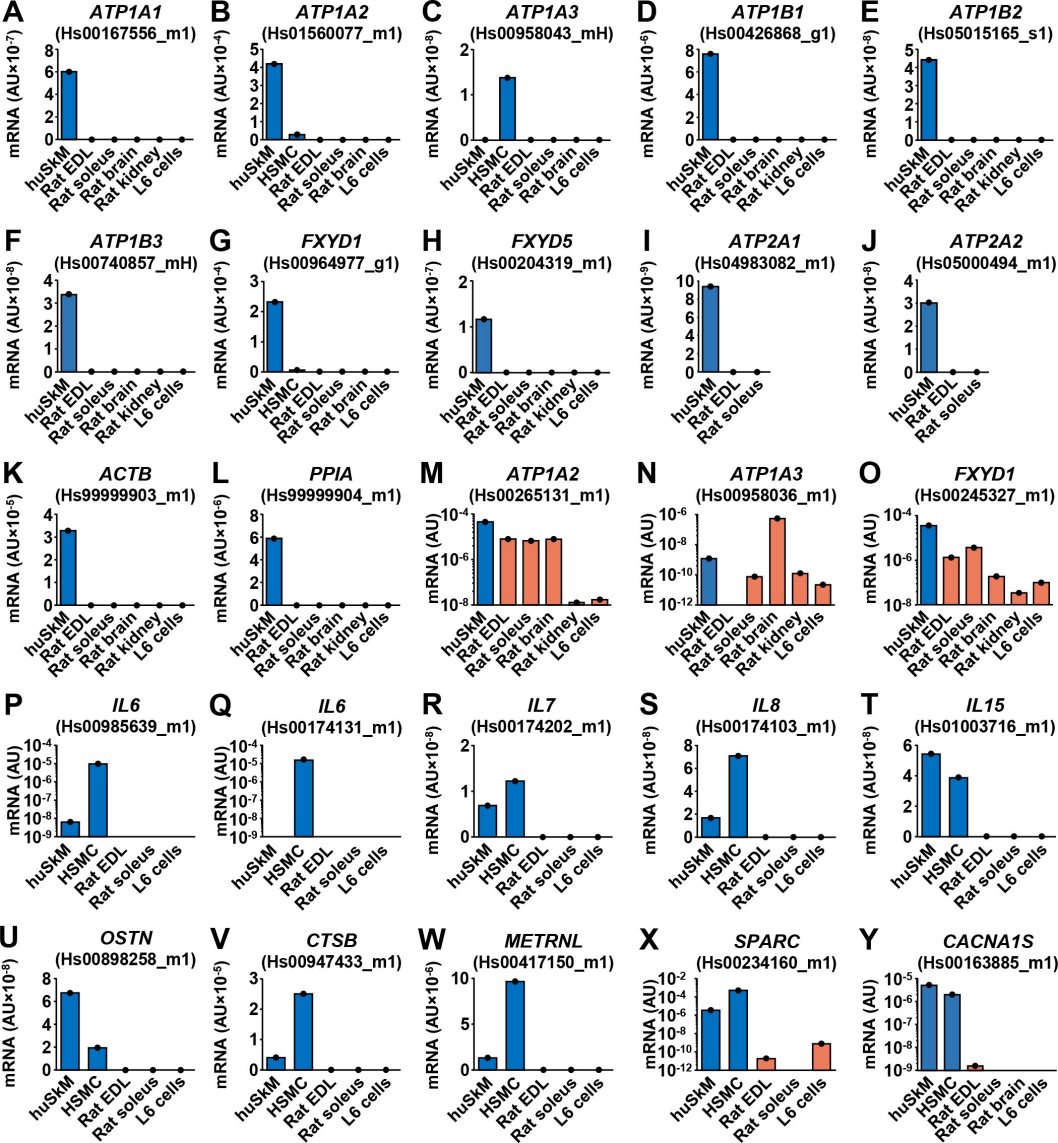
Validation of qPCR assays for estimation of gene expression in innervated human myotubes. (A-Y) Gene expression assays (see Methods) were validated by performing qPCR on human skeletal muscle (huSkM, *m*. *semitendinosus*), human skeletal muscle cells (HSMC, myotubes), and a panel of rat tissues (*m*. *extensor digitorum longus* (EDL) and *m*. *soleus* muscles, brain, and kidney) and L6 skeletal muscle cells. Each tissue or cell sample was measured in duplicate. Gene symbols: the same as in Figs 1 and 5. Endogenous control: 18S rRNA (18S).

Co-cultures contain the muscle (human) and the nervous (rat) components. To estimate gene expression in human myotubes and not in rat neural cells, human-specific gene expression assays were needed. If several commercial human gene expression assays with good target coverage were available we aimed to choose those that bind to regions with the lowest level of sequence similarity between the human and rat transcripts. Candidate assays were then used for qPCR on a panel of rat and human tissue and cell samples (Fig 2). Some human gene expression assays robustly detected rat transcripts (Fig 2M–2O), but only those that produced no amplification in rat samples or were highly selective for human transcripts (Fig 2X and 2Y) were used for analysis of gene expression in innervated myotubes.

### Effect of innervation on the ion transport machinery in human myotubes

**Fig 3:**
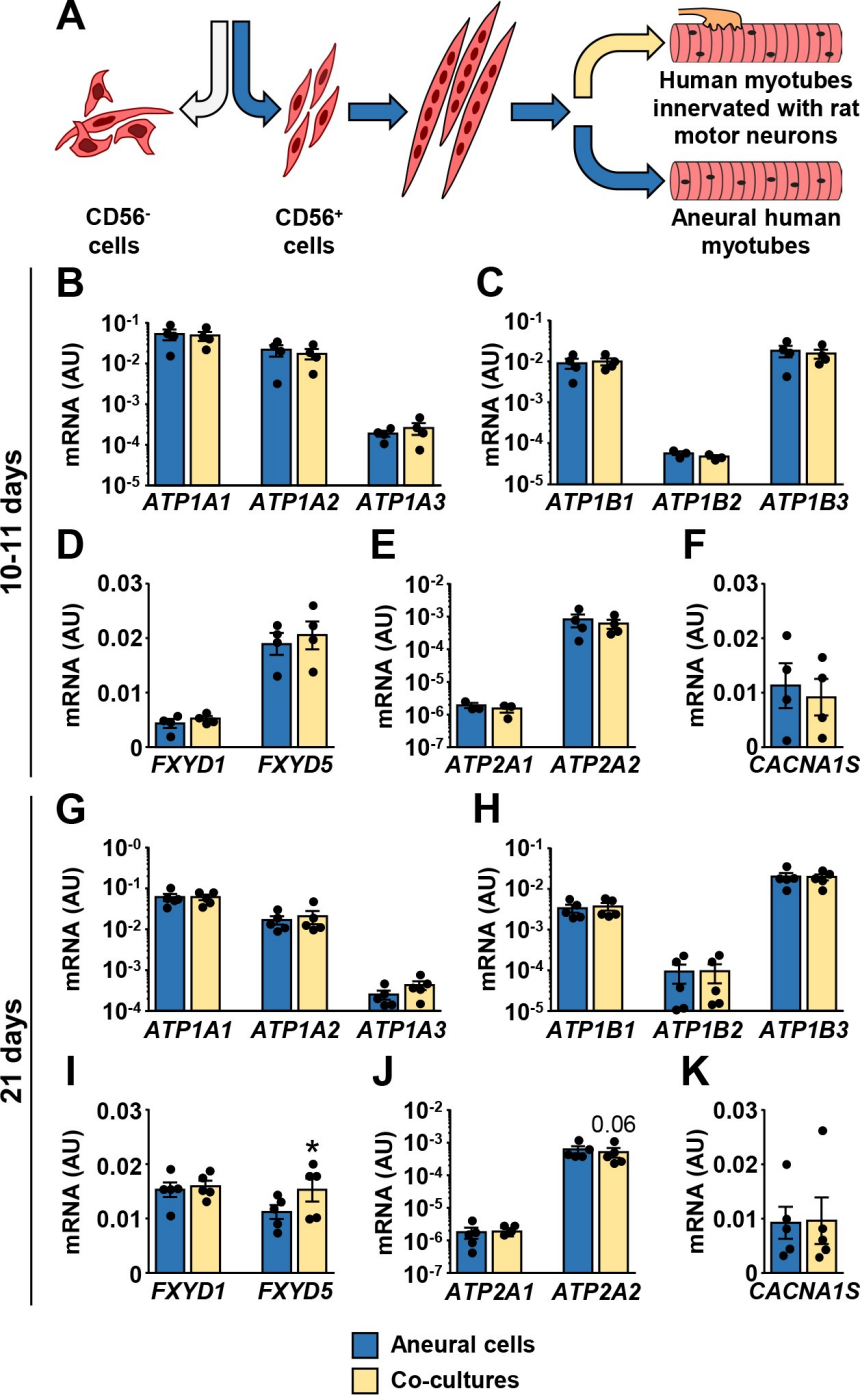
Effect of innervation on mRNA expression of ion transport machinery in human myotubes. (A) CD56^+^ cells were grown aneurally or co-cultured with the embryonic rat spinal cord explants for either 10–11 days (B-F) or 21 days (G-K). (B-K) qPCR was used to estimate gene expression of (B, G) NKAα1–3 (*ATP1A1-3*), (C, H) NKAβ1–3 (*ATP1B1-3*), (D, I) *FXYD1* and *FXYD5*, (E, J) SERCA1 (*ATP2A1*) and SERCA2 (*ATP2A2*), and (F, K) *CACNA1S*. qPCR was performed with human-specific gene expression assays, normalized to geometric mean of ACTB and PPIA endogenous controls. Results are means ± SEM (n = 3–5), *P<0.05—aneural cells vs. co-cultures.

Human skeletal muscle cells (CD56^+^) were differentiated into myotubes and cultured in the presence or absence of the embryonic rat spinal cord explants (Fig 3A) for 10–11 days or 21 days. Co-cultured myotubes started to contract after 7–10 days (S1 Video). Cyclophilin and β-actin (ACTB) were used as endogenous controls to estimate gene expression in innervated myotubes. The mRNA expression of NKAα1–3 (Fig 3B and 3G), NKAβ1–3 (Fig 3C and 3H), and FXYD1 (Fig 3D and 3I) was similar between innervated and aneural myotubes in the short-term (10–11 days) and the long-term (21 days) cultures. FXYD5 mRNA was slightly increased in the long-term co-cultures (Fig 3I). SERCA1, SERCA2 (Fig 3E and 3J), and DHPRα_1S_ (Fig 3F and 3K) mRNA were unaltered by innervation.

**Fig 4:**
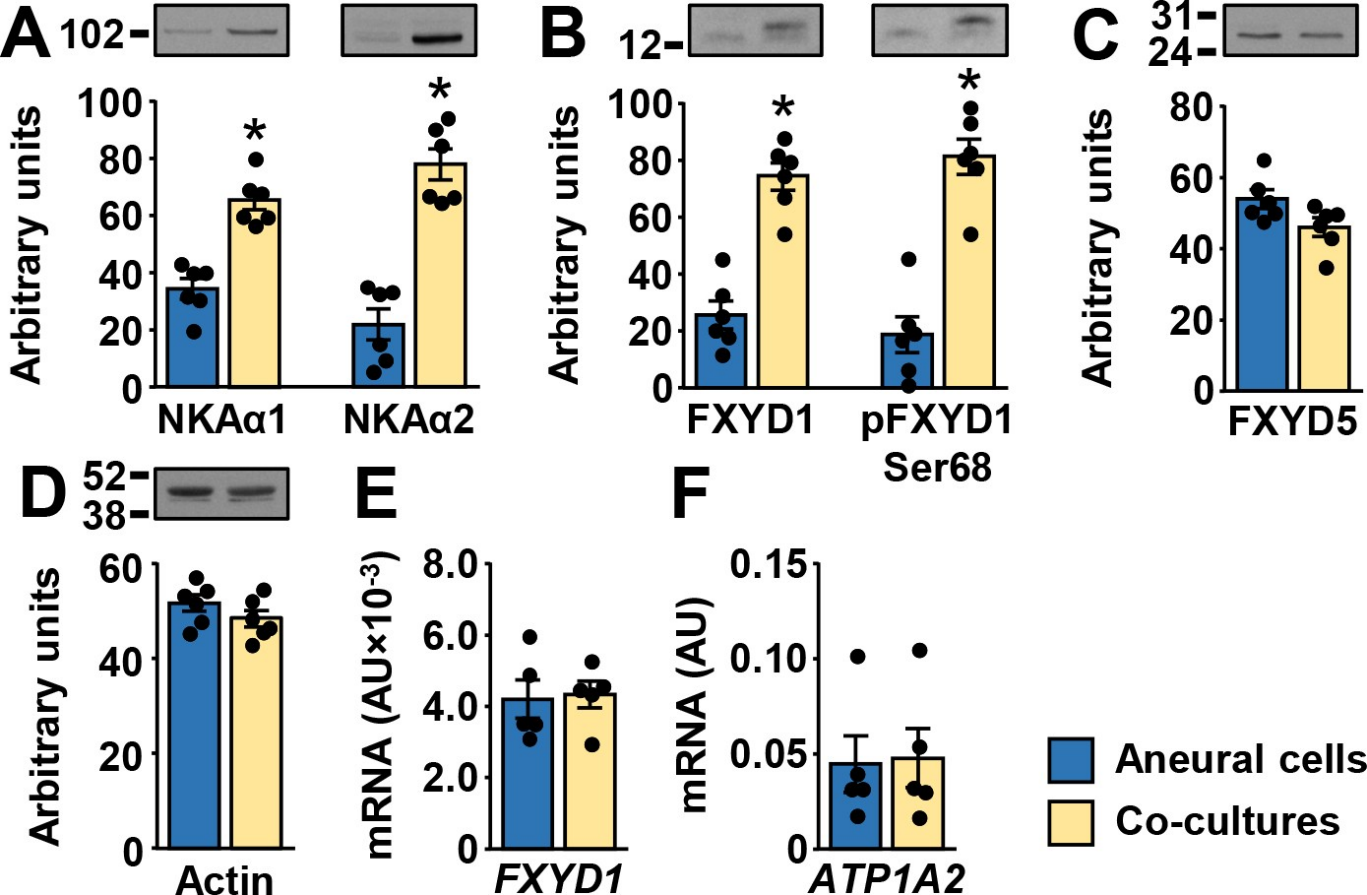
Effect of innervation on the protein abundance of NKA subunits, FXYD1, and FXYD5 in human myotubes. (A-D) Immunoblotting was used to estimate protein abundance of (A) NKAα1 and NKAα2, (B) FXYD1 and pFXYD1^Ser68^, (C) FXYD5, and (D) actin. (E,F) qPCR was performed with gene expression assays for (E) FXYD1 (Hs00245327_m1) and (F) NKAα2 (Hs00265131_m1) that detect human and rat transcripts. Results are means ± SEM (n = 5–6), *P<0.05—aneural cells vs. co-culture.

The abundance of NKAα1 and NKAα2 protein was higher in co-cultures (Fig 4A). NKAα3 could not be detected reliably with immunoblot. The abundance of the total and phosphorylated (Ser^68^) FXYD1 protein was markedly higher in co-cultures (Fig 4B). FXYD5 (Fig 4C) and actin (Fig 4D) protein levels were similar between co-cultured and aneural myotubes. Primary antibodies used in these analyses were not species-specific, meaning that rat neural proteins might have been detected with immunoblotting. However, NKAα2 and FXYD1 gene expression assays, which detect human and rat transcripts, did not show differences in mRNA expression between aneural and co-cultured cells (Fig 4E and 4F), indirectly suggesting contribution of NKAα2 and FXYD1 proteins from rat cells was relatively low.

### Effect of innervation on the expression of myokines in human myotubes

**Fig 5:**
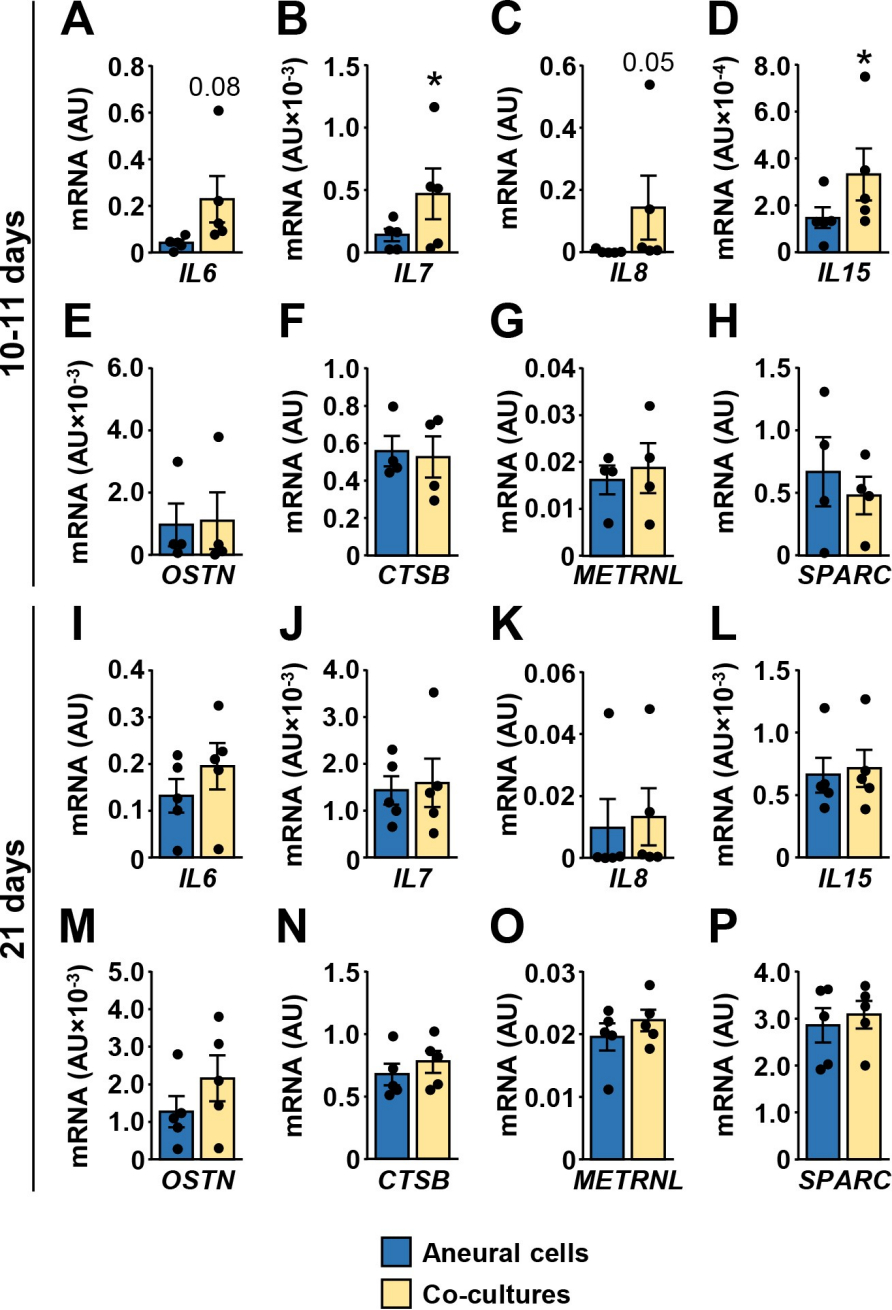
Effect of innervation on the expression of myokines in human myotubes. CD56^+^ cells were grown aneurally or were co-cultured with the embryonic rat spinal cord for either 10–11 days (A-H) or 21 days (I-P). (A-P) qPCR was used to estimate gene expression of (A, I) interleukin-6 (*IL6*), (B, J) interleukin-7 (*IL7*), (C, K) interleukin-8 (*IL8*), (D, L) interleukin-15 (*IL15*), (E, M) musclin (*OSTN*), (F, N) cathepsin B (*CTSB*), (G, O) meteorin-like protein (*METRNL*), and (H, P) *SPARC*. qPCR was performed with human-specific gene expression assays, normalized either to ACTB (A-H) or to geometric mean of ACTB and PPIA endogenous controls (I-P). Results are means ± SEM (n = 4–5), *P<0.05 –aneural cells vs. co-cultures.

Myokines are muscle-derived cytokines and other peptides whose expression and secretion from skeletal muscle fibers is regulated by contractions [49, 50]. To determine whether innervation-induced contractions altered expression of myokines, we measured mRNA levels of prototypical myokines IL-6, IL-7, IL-8, and IL-15 [49, 50] (Fig 5). In addition, we measured expression of musclin (aka osteocrin) [51–53], cathepsin B [54], meteorin-like protein (aka subfatin) [55], and secreted protein acidic and rich in cysteine (SPARC) [56] (Fig 5), which have all been established as myokines more recently. At 10–11 days of innervation, expression of IL-6, IL-7, IL-8, and IL-15 mRNA (Fig 5A–5D) was upregulated or tended to be upregulated, while musclin, cathepsin B, meteorin-like protein and SPARC mRNA levels were unaltered (Fig 5E–5H). At 21 days differences between innervated and aneural myotubes were not significant (Fig 5I–5P).

### Effect of α-bungarotoxin and tubocurarine on the expression of NKA subunits, FXYD1, and FXYD5

**Fig 6:**
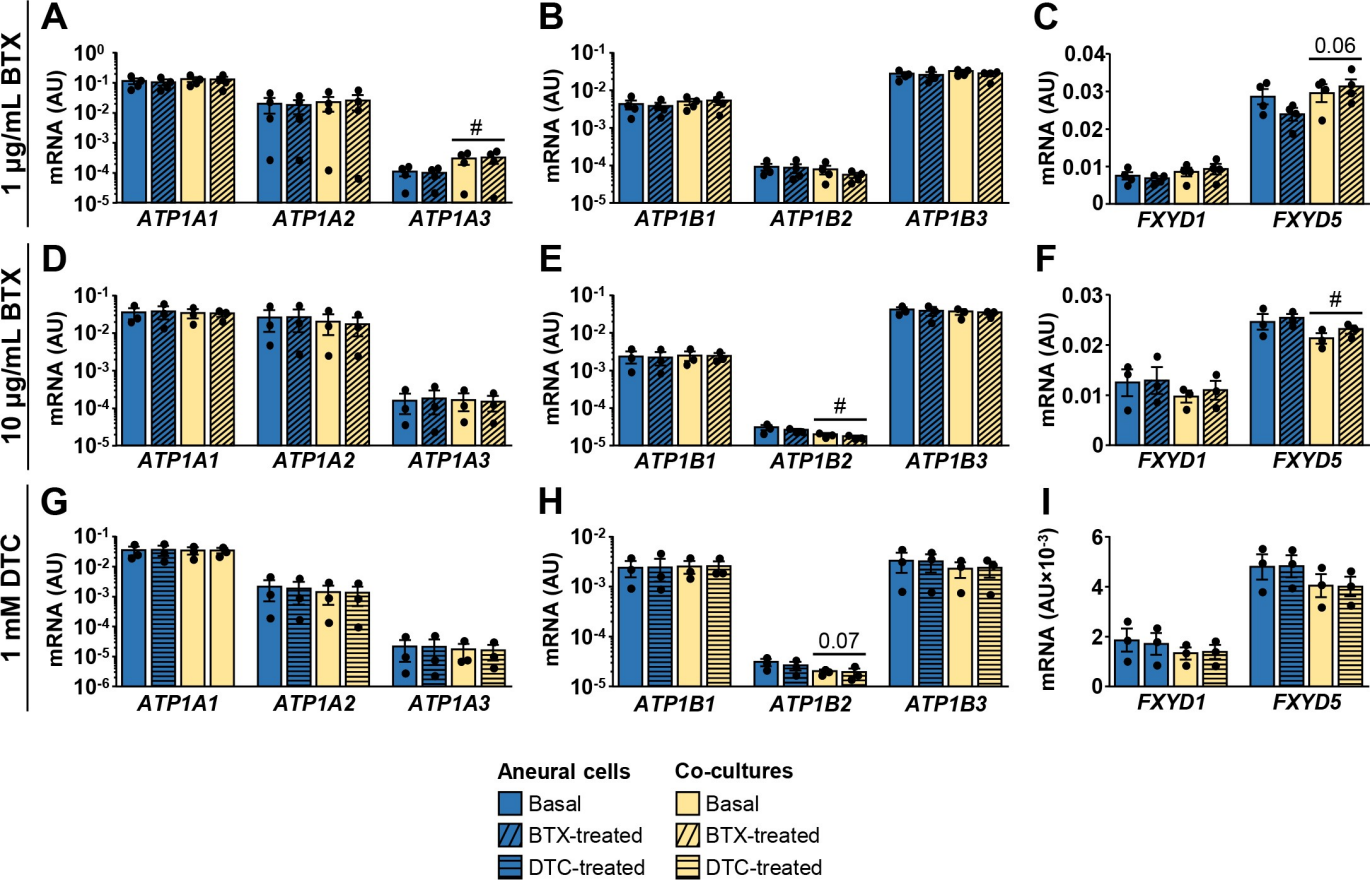
Effect of α-bungarotoxin and tubocurarine on expression of NKA subunits, FXYD1, and FXYD5. 21-day old co-cultures and aneural controls were treated with 1 μg/mL α-bungarotoxin (BTX) (A-C), 10 μg/mL α-bungarotoxin (D-F) or 1 mM d-tubocurarine (DTC) (G-I) for 24 h. (A-I) qPCR was used to estimate gene expression (geometric mean of ACTB and PPIA was used for normalization of results) of (A,D,G) NKAα1–3 (*ATP1A1-3*), (B,E,H) NKAβ1–3 (*ATP1B1-3*), or (C,F,I) *FXYD1* and *FXYD5*. Human-specific gene expression assays were used. Results are means ± SEM (n = 3–4), #*P*<0.05 –aneural cells vs. co-cultures. Basal aneural cell and co-culture samples for 10 μg/mL α-bungarotoxin and 1 mM d-tubocurarine are the same, but were measured on separate occasions.

Acetylcholine and nAChR were implicated in regulation of NKA in skeletal muscle [5, 57–62]. We therefore examined whether suppression of neuromuscular transmission with pharmacological antagonists of nAChR alters expression of NKA subunits or FXYDs. To suppress neuromuscular transmission, the long-term co-cultures (21-day old) and their aneural controls were treated for 24 h with α-bungarotoxin (1 μg/ml or 10 μg/ml), an irreversible nAChR antagonist, and tubocurarine (1 mM), a reversible nAChR antagonist. Effect on contractions was variable between donors, meaning that contractions stopped completely in some cultures, while others retained some contractile activity, consistent with our previous observations in co-cultures treated with nAChR antagonist rocuronium [27]. Overall mRNA levels of NKA subunits, FXYD1, and FXYD5 remained unaltered by α-bungarotoxin (Fig 6A–6F) or tubocurarine treatment (Fig 6G–6I).

### Effect of EPS on ion transport machinery in human myotubes

**Fig 7:**
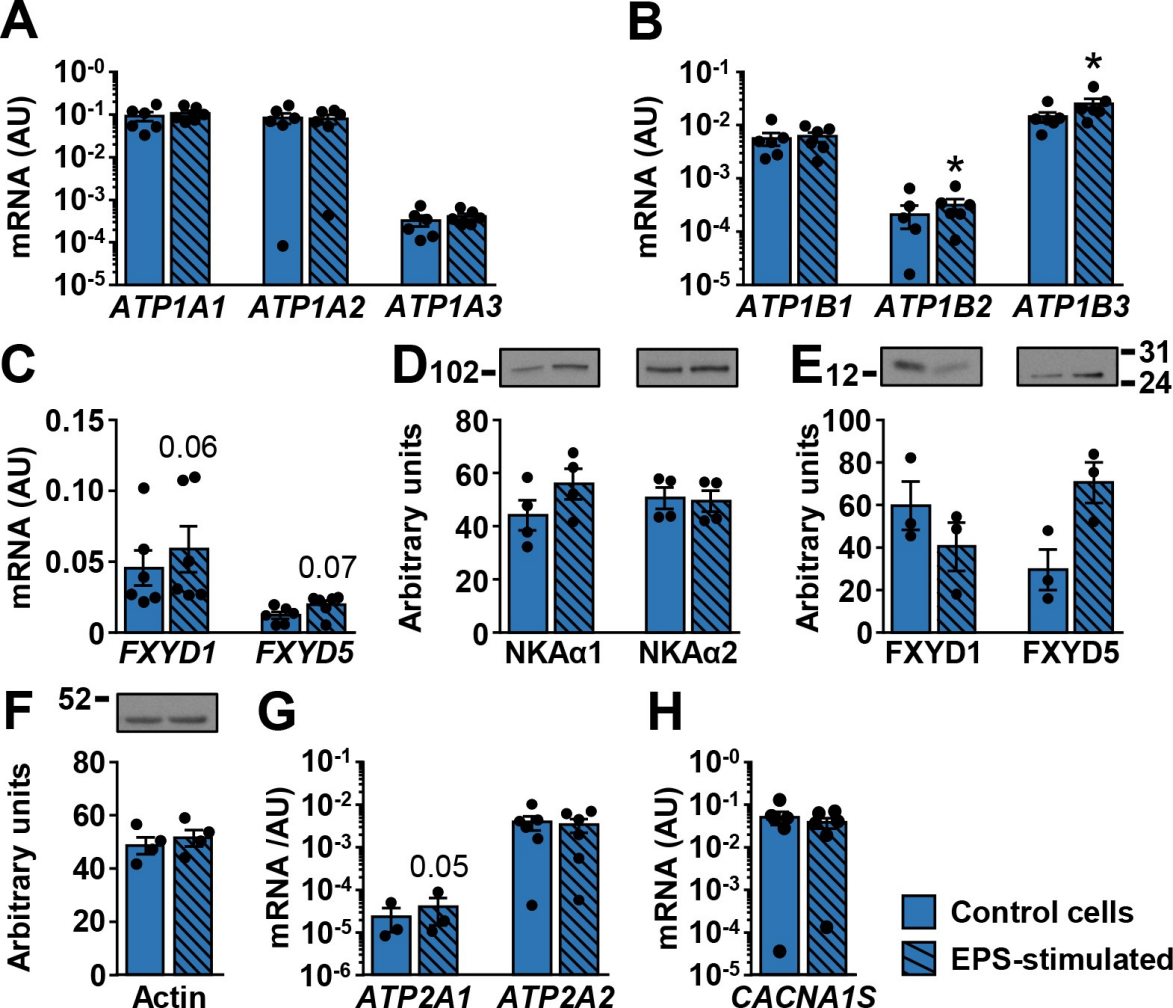
Effect of EPS on the ion transport machinery in human myotubes. qPCR was used to estimate gene expression of (A) NKAα1–3 (*ATP1A1-3*), (B) NKAβ1–3 (*ATP1B1-3*), (C) *FXYD1* and *FXYD5*, (G) SERCA1 (*ATP2A1*) and SERCA2 (*ATP2A2*), and (H) *CACNA1S*. A geometric mean of ACTB and PPIA was used for normalization of gene expression. Immunoblotting was used to estimate protein abundance of (D) NKAα1 and NKAα2, (E) FXYD1 and FXYD5, and (F) actin. Results are means ± SEM (n = 3–6), *P<0.05 vs non-stimulated (Control) cells.

In addition to innervation, in vitro contractions can be obtained by exposing human myotubes to EPS [29, 30, 63]. To test whether electrically-stimulated contractions modulate expression of NKA subunits and FXYDs, myotubes were exposed to EPS for the last 48 h of differentiation. EPS did not alter mRNA expression of NKAα1–3 (Fig 7A) and NKAβ1 (Fig 7B), while NKAβ2 and NKAβ3 were increased by EPS (Fig 7B). EPS tended to increase FXYD1 (*P* = 0.06) and FXYD5 (*P* = 0.07) mRNA. The protein abundance of NKAα subunits (Fig 7D) was similar between control and EPS-treated myotubes. The abundance of FXYD1 was lower and the abundance of FXYD5 was higher in EPS-treated myotubes than in control cells (Fig 7E), but the differences did not reach the level of statistical significance. EPS did not alter the abundance of actin (Fig 7F). EPS increased mRNA expression of SERCA1 (Fig 7G), but had no effect on the expression of SERCA2 (Fig 7G) and DHPRα_1S_ (Fig 7H).

### Relative gene expression levels of NKA subunits, FXYD1, and FXYD5 in different human skeletal muscle cell models

**Fig 8:**
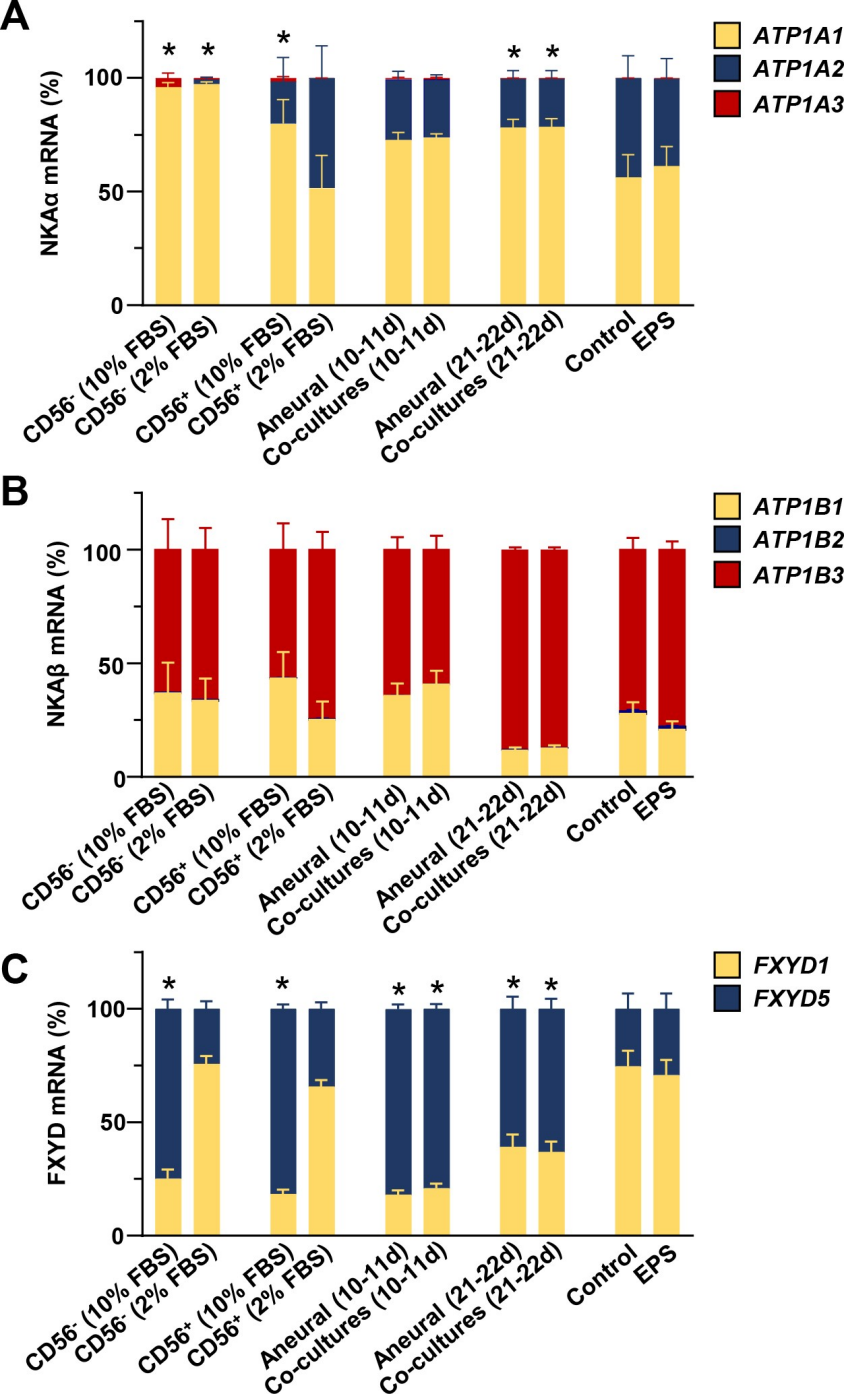
Relative expression levels of NKA subunits, FXYD1, and FXYD5 in different human skeletal muscle cell models. (A-C) Relative mRNA levels of (A) NKAα1–3 (*ATP1A1-3*), (B) NKAβ1–3 (*ATP1B1-3*), (C) *FXYD1* and *FXYD5* in different models were calculated based on results in Figs 1, 3, 6 and 7. CD56^-^ and CD56^+^ cells were grown in the presence of 10 or 2% FBS as described in Fig 1. Co-cultures and aneural control cultures were grown in the presence of 10% FBS for 10–11 days (Fig 3) or 21 days (Fig 3) or 22 days (Fig 6). EPS was performed during the last 2 days of differentiation in the presence of 2% FBS (Fig 7). Expression level of NKAα1, NKAβ1, and FXYD1 mRNA were arbitrarily chosen as 1. Results are means ± SEM (n = 4–12 (co)cultures), *P<0.05 vs. CD56^+^ (2% FBS).

A major aim of the study was to establish whether gene expression of NKA subunits in co-cultured and EPS-treated myotubes more closely resembles expression pattern in skeletal muscle tissue. Based on PCR results (Figs 1, 3, 6 and 7) we calculated the mRNA ratios between NKA subunits and FXYDs in different differentiation stages and human myotube models (Fig 8). The NKAα2 isoform was present in very low concentrations in CD56^-^ cells. In contrast, it represented almost 50% of NKAα mRNA in myotubes cultured under low serum (2% FBS) conditions (Fig 8A). In the long-term co-cultures and aneural controls, which were grown in the presence of 10% FBS, fraction of NKAα2 mRNA was lower than in aneural myotubes (CD56^+^ cells) cultured in the presence of 2% FBS. The innervation or EPS did not increase the fraction of NKAα2 mRNA.

NKAβ3 mRNA was the most abundant NKAβ mRNA under all experimental conditions and differentiation, *de novo* innervation, or EPS did not significantly alter expression pattern of NKAβ isoforms (Fig 8B). Reduction of serum concentration from 10% to 2% markedly increased the FXYD1-to-FXYD5 ratio in CD56^+^ and CD56^-^ cells (Fig 8C). FXYD1 was also prominently expressed in myotubes from the EPS experiment, which were grown in the presence of 2% FBS. Innervation or EPS had no effect on the relative FXYD1 and FXYD5 mRNA levels.

## Discussion

Here we show that innervation or EPS of cultured myotubes did not lead to major changes in expression pattern of NKA subunits, FXYD1, and FXYD5. Conversely, differentiation of human skeletal muscle cells into myotubes under low serum conditions (2%) markedly upregulated NKAα2 as well as several major myogenic markers. We also show that low serum promoted upregulation of FXYD1 mRNA and downregulation of FXYD5 mRNA although changes in gene expression were not always paralleled with significant changes in the protein abundance. In contrast to the NKAα subunits and FXYDs, mRNA expression of the NKAβ subunits was relatively stable under low serum conditions and differentiation into myotubes, suggesting they are regulated via distinct regulatory mechanisms.

A major novelty of our study is that we demonstrate upregulation of FXYD1 mRNA and protein during differentiation of human skeletal muscle cells. Indeed, while FXYD1 plays a major role in skeletal muscle physiology, investigation of the underlying molecular mechanisms has been hampered by lack of appropriate skeletal muscle cell models. For instance, in rat L6 skeletal muscle cells, which is one of the most commonly used *in vitro* models to study skeletal muscle, FXYD1 is below detection limit [64]. Expression of FXYD1 in cultured skeletal muscle cells was therefore considered to be too low to be accessible to experimental manipulation. We show that FXYD1 can be studied in cultured human myotubes, which opens interesting new opportunities to examine its functional role and regulation in skeletal muscle.

The abundance of the total and the phosphorylated (at Ser^68^) FXYD1 was also increased in co-cultures at 21 days. Although cellular localization of FXYD1 in co-cultures should be further examined using immunocytochemistry, it is tempting to speculate about functional consequences of altered FXYD1 levels. Indeed, while unphosphorylated FXYD1 suppresses NKA activity by decreasing its affinity for intracellular Na^+^ (increasing *K*_*1/2*,*Na*_), phosphorylation of Ser^63^ or Ser^68^ reverses this effect, thus activating NKA [10, 11, 17–19]. Increased levels of phosphorylated FXYD1 therefore indirectly suggest that NKA activity in innervated contracting myotubes was higher than in aneural (non-innervated) quiescent myotubes. Since contractions displace Na^+^ and K^+^ ions across the membrane, NKA activation, which opposes this displacement, would make physiological sense. Further, increased abundance of phosphorylated FXYD1 is consistent with observations that *ex vivo* skeletal muscle contractions can sometimes, although not always [65], stimulate phosphorylation of FXYD1 [66]. Although the link between FXYD1 and contraction of innervated myotubes still needs to be examined, it is important to note that AMP-activated protein kinase, which is activated during muscle contractions [67], can regulate FXYD1 phosphorylation as well as the abundance of the total FXYD1 in skeletal muscle [68] (reviewed in: [69]).

As assessed by mRNA measurements, human skeletal muscle expresses primarily NKAα2 (~87% of NKAα transcripts) and NKAα1 (~13% of NKAα transcripts) [23], consistent with data from rat muscles, which indicated that NKAα2 represents ~60–90% of NKAα subunits [12, 70–73]. NKAα2 was robustly expressed in differentiated CD56^+^ myotubes (~48% of NKAα transcripts), but NKAα1 remained the predominant NKAα isoform (>50% of NKAα transcripts) in cells from most donors (Fig 8), consistent with observations in our recent study [24]. Innervation or EPS did not further increase NKAα2 mRNA, indicating neither formation of functional neuromuscular junctions nor contractions are essential for regulation of NKAα2 expression. Based on our results and previous work [74] we can assume that a major trigger for upregulation of NKAα2 is deprivation of serum. Indeed, the innervated myotubes, which were grown in the presence of 10% FBS, had lower relative NKAα2 mRNA expression (mean ~20%) than myotubes, which were differentiated under low serum conditions (Fig 8). Similarly, FXYD1 was more prominently expressed than FXYD5 in all cells grown in the presence of 2% FBS, while the reverse was observed in the presence of 10% FBS. Under *in vitro* conditions serum concentration therefore seems to be a more important regulator of NKAα and FXYD expression than innervation or contractile activity.

Innervation of cultured myotubes stimulates development of T-tubular system, which is immature and rudimentary in aneural myotubes [25]. Since NKAα2 is located mainly in the T-tubular system [75], we expected innervation would lead to upregulation of NKAα2 in co-cultures due to formation of T-tubules [76]. Upregulation in contracting myotubes was also expected because physical activity increases NKA abundance [77], while physical inactivity decreases it [78]. Indeed, at 21 days co-cultured myotubes were contracting for ~10–14 days, while aneural myotubes were quiescent. However, abundance of the muscle-specific isoforms of creatine kinase and phosphorylase progressively increases up to 42 days of co-culture [79]. Also, while the 21-day time-point was chosen to assess NKA expression because deeper T-tubules first appear after 3 weeks of co-culture, T-tubular system continues to develop for several weeks [25]. The postnatal increase in NKA content in mouse and rat skeletal muscle occurs in parallel with the development of the T-tubular system [80, 81]. Thus, 21 days of co-culture might not have been sufficient to observe alterations of NKAα2 expression, which is predominantly destined for localization in T-tubules. Consistent with this notion, we found that expression of DHPRα_1S_, which is located in T-tubules [47, 76], was unaltered by innervation.

Co-cultures displayed increased protein abundance of NKAα1 and NKAα2 protein. Total and phosphorylated FXYD1^Ser68^ were also markedly increased. The antibodies that we used in this study were not human-specific, which means that the apparent increase in protein levels could reflect admixture of neural (rat) proteins rather than changes in muscle protein content. However, gene expression assays for NKAα2 and FXYD1 that detect human and rat transcripts produced the same result as human-specific assays, indirectly suggesting contamination with rat transcripts was not extensive. This idea could be verified by measuring NKA activity in the presence of different ouabain concentrations. Indeed, rat NKAα1 is markedly less sensitive to ouabain than NKAα2 and NKAα3, which means that it can be used to distinguish between the contribution of rat NKAα1 and rat NKAα2 and/or NKAα3 [58, 82, 83]. A corollary to this is that ouabain could be used in co-cultures to evaluate the contribution of human (muscle) NKAα subunits, which are highly and similarly sensitive to its inhibitory actions [84], and the ouabain-resistant rat NKAα1 subunits [85–88] from the spinal cord explants. While cellular distribution of NKA subunits and FXYDs in co-cultures will have to be further examined, it is tempting to speculate that innervation regulates these proteins posttranscriptionally, which would explain discrepancies between mRNA and protein levels. Perhaps innervation enhances translation or suppresses proteolysis, thus leading to an increase in protein levels of NKA and FXYD1 without alterations in mRNA levels. Consistent with this notion, actinomycin D, which inhibits RNA synthesis, did not prevent a postnatal increase in the NKA density in rat soleus muscle, although it suppressed muscle growth [80].

In our study NKA gene expression was unaltered by innervation, which appears to contradict classical denervation/reinnervation experiments, which showed that innervation increases NKA content in skeletal muscle [7, 8]. However, reinnervation of mature skeletal muscle fibers, which were fully functional before they had been denervated, cannot be directly compared to *de novo* innervation of immature myotubes, which do not contract and start to develop more mature characteristics only once innervated [25]. Notably, mRNA expression of IL-6, IL-7, IL-8, and IL-15 was increased or tended to be increased at 10–11 days of co-culture (up to 4 days of contractions), while their expression returned to normal after 21 days in co-culture (up to 14 days of contractions). This result suggests that innervation and contractile activity *in vitro* promote myokine expression acutely but not chronically, consistent with observations that endurance training suppresses IL-6 expression (reviewed in: [49, 50]). Differences in transcriptional responses between myokines and NKA subunits indicate that the level of contractile activity was likely not sufficient to affect NKA expression in innervated myotubes. Similarly, EPS did not increase expression of NKAα subunits, although it increased mRNA expression of NKAβ2 and NKAβ3. Collectively, our results suggest that innervation and EPS are not sufficient to profoundly alter expression pattern of NKA subunits and FXYDs in cultured myotubes. Differentiation process and serum concentration therefore seem to play a decisive role in regulation of their expression in cell culture.

At the neuromuscular junction nAChR is involved in regulation of NKA [58, 59], and α-bungarotoxin, which irreversibly inhibits the nAChR, blocks activation of NKA by acetylcholine [62]. In our study, α-bungarotoxin or tubocurarine did not alter expression of NKA subunits, FXYD1, and FXYD5 in innervated myotubes. We measured gene expression not only in the perisynaptic region but in whole homogenates, which may explain why inhibition of the nicotinic receptor had no effect. Although we did not assess the stability of NKA and FXYD mRNA in co-cultures and 24 h functional denervation with α-bungarotoxin might have been too short to alter their levels, we can conclude that acute blockade of the neuromuscular transmission does not produce alterations in NKA or FXYD gene expression.

One of the aims of the study was to establish whether myotubes co-cultured with the embryonic rat spinal cord display NKA and FXYD expression pattern that more closely resembles their expression in muscle fibers *in vivo* than aneurally cultured myotubes. While differentiation of myoblasts into myotubes upregulated NKAα2 and FXYD1 mRNA, no further increases were seen in innervated cultures despite contractions. However, we did not determine cellular or subcellular localization of NKA subunits and FXYDs. Importantly, our recent study showed that distribution of MyHC and actin differs between aneural and innervated myotubes [30], indicating that structural maturation of myotubes under *in vitro* conditions is not necessarily associated with marked changes in mRNA and/or protein levels. Further, since we analysed co-culture homogenates we cannot exclude the possibility that alterations in innervated myotubes are at least partially obscured by those of non-innervated myotubes and non-muscle cells that are present in these cultures. While immunocytochemistry could help to resolve these questions, it is clear that co-culturing of myotubes with the embryonic rat spinal cord for up to 3 weeks does not lead to NKA and FXYD expression pattern that mimics conditions in human skeletal muscle tissue.

In conclusion, our results indicate that during the early stages of *de novo* innervation, motor neurons do not directly regulate gene expression of NKA subunits and FXYD1. Also, EPS of myotubes does not result in increased NKAα or FXYD1 gene expression or protein abundance. Taken together, our results suggest that concentration of serum in cell culture medium is a major factor regulating expression pattern of NKAα subunits and FXYD1 in cultured myotubes. It will be important to determine which serum components and transcription factors are involved in regulation of serum-dependent NKAα and FXYD1 expression. Finally, our study demonstrates that cultured human skeletal muscle cells can be used to study FXYD1 and FXYD5, which opens new possibilities for examination of their functions in skeletal muscle.

## Supporting information

S1 Video 3-week old contracting myotubes. (MP4)

S1 Raw images (PDF)

S1 Data Supporting dataset for Figs 1–8. (XLSX)
