# Supplementary material for: Effect of differentiation, de novo innervation, and electrical pulse stimulation on mRNA and protein expression of Na+,K+-ATPase, FXYD1, and FXYD5 in cultured human skeletal muscle cells
Source: PLoS One. 2021 Feb 26;16(2):e0247377. doi: 10.1371/journal.pone.0247377 (PMC7909653; doi:10.1371/journal.pone.0247377)
Supplement: S1 Raw images — (PDF) [file pone.0247377.s002.pdf]

S1_raw_images

Effect of differentiation, *de novo* innervation, and electrical pulse stimulation on mRNA and protein expression of Na^+,K^+-ATPase, FXYD1, and FXYD5 in cultured human skeletal muscle cells

Vid Jan[1], Katarina Miš[1], Natasa Nikolic[2], Klemen Dolinar[1], Metka Petrič[1], Andraž Bone[1], G. Hege Thoresen[2,3], Arild C. Rustan[2], Tomaž Marš[1], Alexander V. Chibalin[4,5], Sergej Pirkmajer[1,*]

[1]Institute of Pathophysiology, Faculty of Medicine, University of Ljubljana, Ljubljana, Slovenia

[2]Section for Pharmacology and Pharmaceutical Biosciences, Department of Pharmacy, University of Oslo, Oslo, Norway

[3]Department of Pharmacology, Institute of Clinical Medicine, University of Oslo, Oslo, Norway

[4]National Research Tomsk State University, Tomsk, Russia

[5]Department of Molecular Medicine and Surgery, Integrative Physiology, Karolinska Institutet, Stockholm, Sweden

*Corresponding author

E-mail: sergej.pirkmajer@mf.uni-lj.si

Immunoreactive bands were visualized on X-ray films using enhanced chemiluminescence and quantified using GS-800 Densitometer and Quantity One 1-D Analysis Software 4.6.8. (Bio-Rad, Hercules, CA, U.S.).

Loading and transfer were evaluated by Ponceau S (0.1% (w/v) in 5% (v/v) acetic acid) staining.

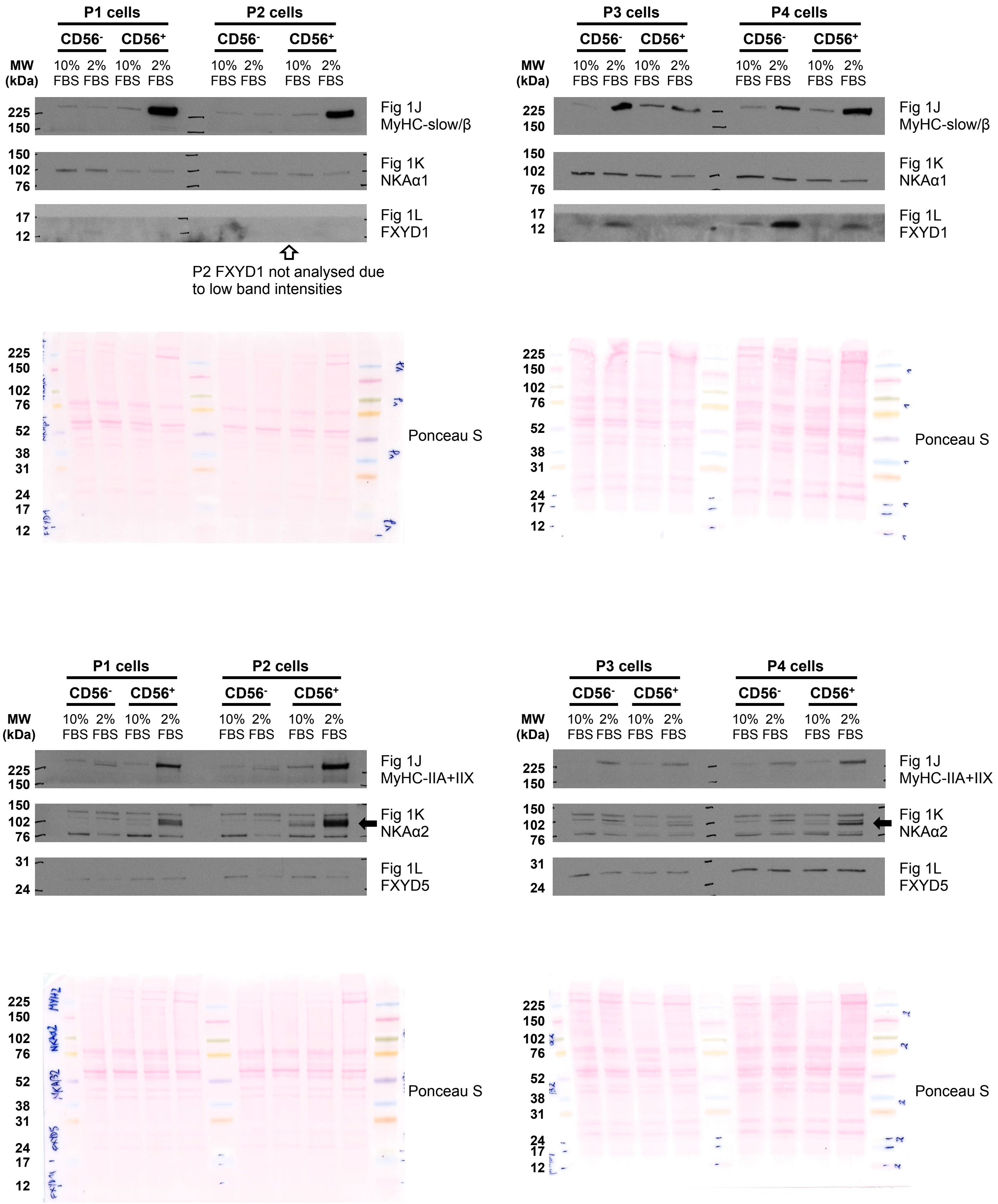

Original uncropped blots presented in Fig 1 (1/2)

Immunoreactive bands were visualized on X-ray films using enhanced chemiluminescence and quantified using GS-800 Densitometer and Quantity One 1-D Analysis Software 4.6.8. (Bio-Rad, Hercules, CA, U.S.).

Loading and transfer were evaluated by Ponceau S (0.1% (w/v) in 5% (v/v) acetic acid) staining.

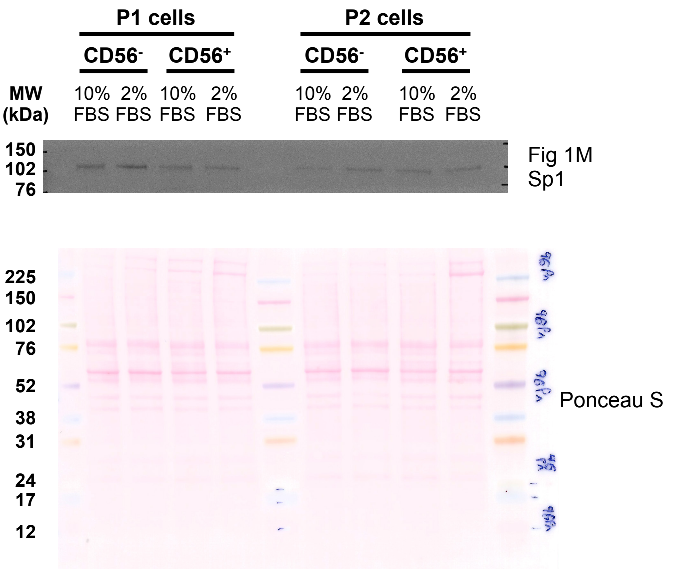

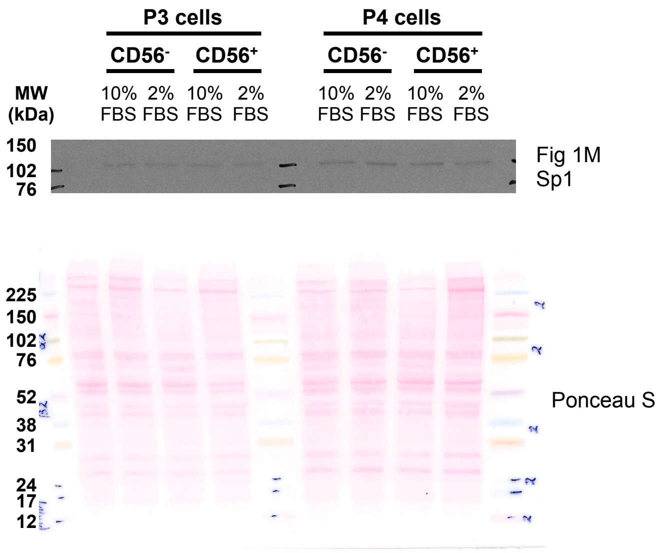

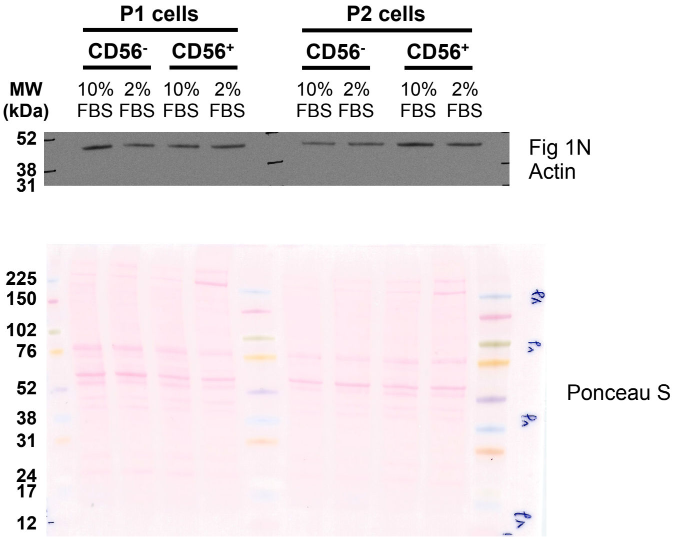

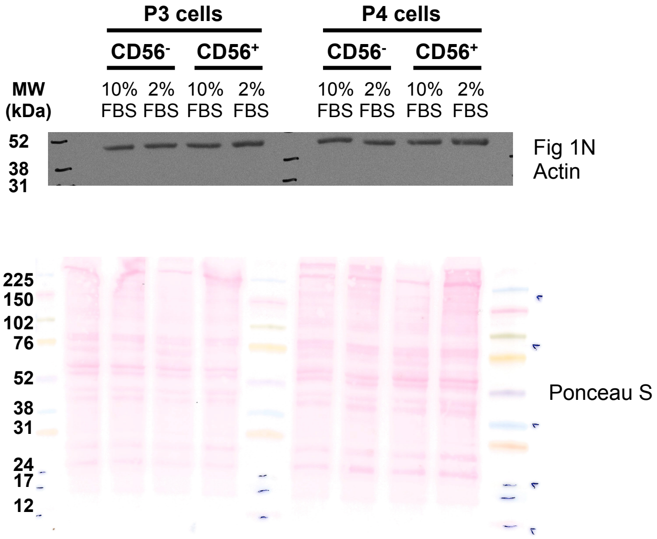

Original uncropped blots presented in Fig 1 (2/2)

Immunoreactive bands were visualized on X-ray films using enhanced chemiluminescence and quantified using GS-800 Densitometer and Quantity One 1-D Analysis Software 4.6.8. (Bio-Rad, Hercules, CA, U.S.).

Loading and transfer were evaluated by Ponceau S (0.1% (w/v) in 5% (v/v) acetic acid) staining.

Legend:

AN: Aneural cells
CO: Co-cultures

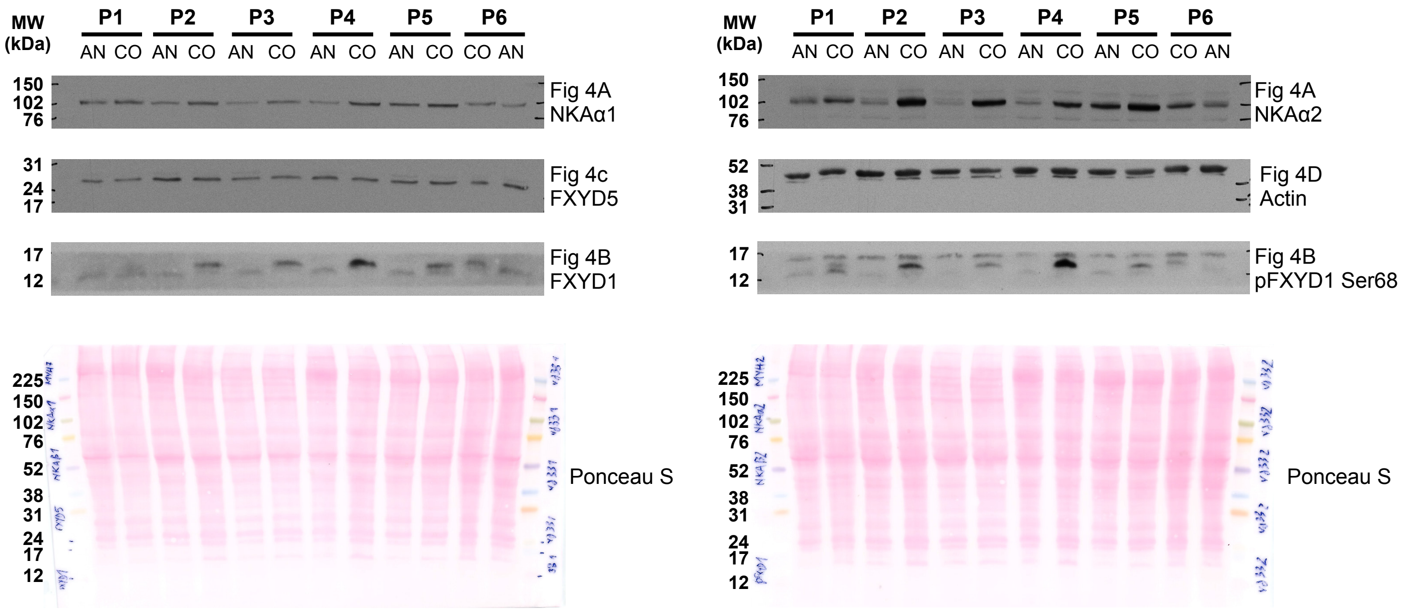

P6 donor samples were accidentally loaded in reverse order.

Original uncropped blots presented in Fig 4

Immunoreactive bands were visualized on X-ray films using enhanced chemiluminescence and quantified using GS-800 Densitometer and Quantity One 1-D Analysis Software 4.6.8. (Bio-Rad, Hercules, CA, U.S.).

Loading and transfer were evaluated by Ponceau S (0.1% (w/v) in 5% (v/v) acetic acid) staining.

Legend:

C: Control cells
E: EPS-stimulated

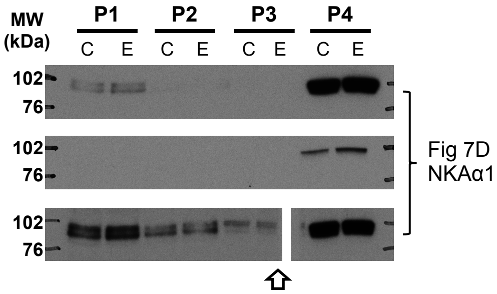

Membrane was accidentally cut and P3 donor samples were re-evaluated on a different membrane.

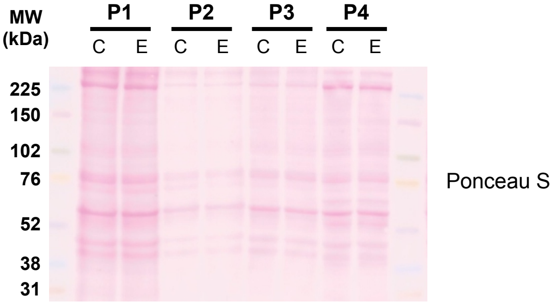

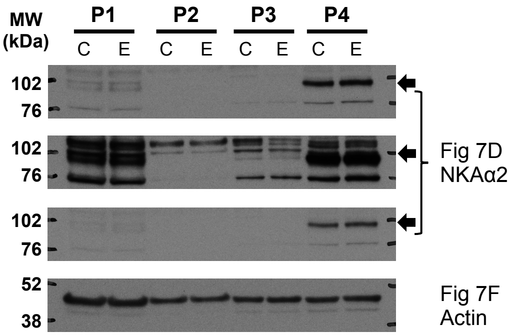

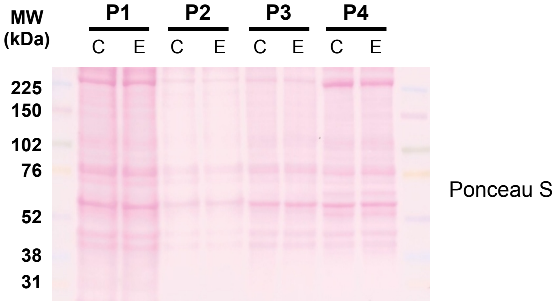

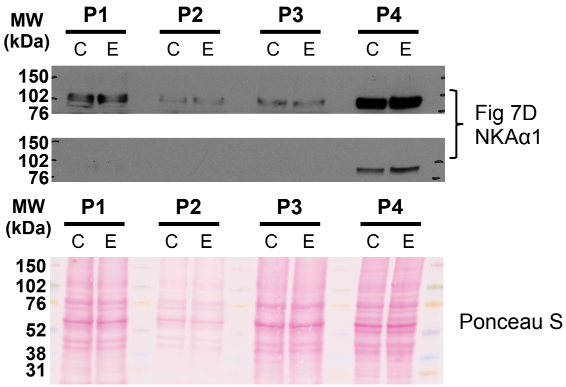

Bands for NKAα1 of P3 donor samples could not be successfully evaluated on the first membrane, so detection was repeated on a fresh membrane.

For P1, P2 and P4 donor samples, band intensity values for NKAα1 were averaged from both membranes.

Due to variability in band intensities between different donors different X-ray film exposures are shown for NKAα1 and NKAα2.

Original uncropped blots presented in Fig 7 (1/2)

Immunoreactive bands were visualized on X-ray films using enhanced chemiluminescence and quantified using GS-800 Densitometer and Quantity One 1-D Analysis Software 4.6.8. (Bio-Rad, Hercules, CA, U.S.).

Loading and transfer were evaluated by Ponceau S (0.1% (w/v) in 5% (v/v) acetic acid) staining.

Legend:

C: Control cells
E: EPS-stimulated

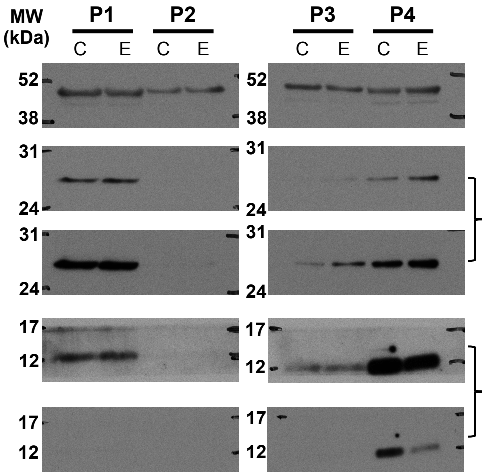

Fig 7F Actin

Fig 7E FXYD5

Fig 7E FXYD1

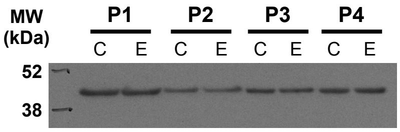

Fig 7F Actin

Due to low band intensities FXYD1 and FXYD5 could not be analysed for P2 donor samples.

Due to high variability in band intensities between different donor samples 2 different X-ray film exposures are shown.

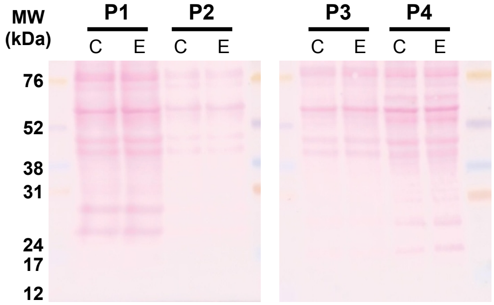

Ponceau S

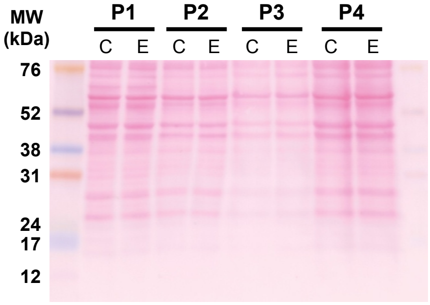

Ponceau S

Original uncropped blots presented in Fig 7 (2/2)
